# *Streptomyces* as a promising biological control agents for plant pathogens

**DOI:** 10.3389/fmicb.2023.1285543

**Published:** 2023-11-14

**Authors:** Shaista Khan, Seweta Srivastava, Arun Karnwal, Tabarak Malik

**Affiliations:** ^1^School of Bioengineering and Biosciences, Lovely Professional University, Phagwara, Punjab, India; ^2^School of Agriculture, Lovely Professional University, Phagwara, Punjab, India; ^3^Department of Biomedical sciences, Jimma University, Jimma, Ethiopia

**Keywords:** agricultural industry, antimicrobial compounds, biocontrol, crop protection, *Streptomyces*, phytopathogens, natural alternatives

## Abstract

Plant diseases caused by pathogenic microorganisms in agriculture present a considerable obstacle, resulting in approximately 30–40% crop damage. The use of conventional techniques to manage these microorganisms, i.e., applying chemical pesticides and antimicrobials, has been discovered to have adverse effects on human health and the environment. Furthermore, these methods have contributed to the emergence of resistance among phytopathogens. Consequently, it has become imperative to investigate natural alternatives to address this issue. The *Streptomyces* genus of gram-positive bacteria is a potentially viable natural alternative that has been extensively researched due to its capacity to generate diverse antimicrobial compounds, such as metabolites and organic compounds. Scientists globally use diverse approaches and methodologies to extract new bioactive compounds from these bacteria. The efficacy of bioactive compounds in mitigating various phytopathogens that pose a significant threat to crops and plants has been demonstrated. Hence, the *Streptomyces* genus exhibits potential as a biological control agent for combating plant pathogens. This review article aims to provide further insight into the *Streptomyces* genus as a source of antimicrobial compounds that can potentially be a biological control against plant pathogens. The investigation of various bioactive compounds synthesized by this genus can enhance our comprehension of their prospective utilization in agriculture.

## 1. Introduction

Rhizosphere microorganisms play a significant role in producing diverse antimicrobial compounds, including metabolites and volatile organic components, aiding plant development and disease prevention (Gong et al., 2022). These compounds, produced by microorganisms, contain inorganic substances like NH_3_, CO_2_, and HCN that can modify the structures of other molecules, such as terpenes, ketones, sulfur-containing, and nitrogen-containing compounds, and aldehydes (Gong et al., 2022). Volatile organic substances possess distinct physical and chemical properties like high vapor pressure, low molecular weight, lipophilic moiety, low boiling point, and ease of movement through the gaseous phase, which facilitate their long-range air and soil diffusion (Gong et al., 2022). Bacteria and fungi have been applied as biofertilizers and biocontrol agents for several years (Qiu et al., 2019). Focusing solely on *in vitro* inhibition overlooks a range of ecological factors that can influence the establishment and persistence of biological control agents in their natural habitats, as highlighted by Kaminsky et al. (2019). When researching microbial methods for controlling plant diseases, the emphasis often falls on specific pathogen-host pairings, raising questions about how effective and applicable these biological control agents are across various plant pathogens and crop varieties, as discussed by LeBlanc (2022). The primary mechanism associated with the effectiveness of biocontrol is the production of multiple antibiotics. Many of these antimicrobial compounds contain enzymes with antifungal and antibacterial properties, such as proteases, lipases, or chitinases, which break down fungal cells and prevent plant-fungal infections. Among these antifungal enzymes, proteases are the most commonly encountered type, as noted by Vurukonda et al. (2018). Similarly, *Streptomyces* strains produce antagonistic compounds, including siderophores, to ward off bacterial infections and exhibit antiviral and antibiofilm properties as well. *Streptomyces* is also responsible for the production of industrially valuable enzymes and a diverse array of biologically active secondary metabolites, which encompass antibiotics, antioxidants, and anticancer agents (Butt et al., 2023). In a broader context, microbial inoculants show great promise as tools for maintaining agricultural sustainability. These microorganisms can enhance plant health and nutrient availability, as emphasized by Qiu et al. (2019). An especially effective approach against *M. incognita* involves combining abamectin and/or emamectin benzoate with *P. lilacinum* and rhizobacteria, as demonstrated by El-Ashry et al. (2021). This approach not only significantly reduces the formation of galls and the reproduction of *M. incognita* but also leads to marked improvements in various tomato growth parameters compared to the control group. The use of various bioagents, including abamectin, holds great promise as a potential antagonistic strategy against phytonematodes in protected agricultural environments, as indicated by El-Ashry et al. (2021).

Plant diseases are classified into two types based on the pathogens that cause them: infectious and non-infectious. Plant diseases that are not infectious do not spread from one plant to another, whereas infectious plant diseases can travel from an infected plant to another healthy plant (Nazarov et al., 2020). Both biotic and abiotic factors can influence plant disease development. Plant illnesses are distinguished by symptoms such as spotting (necrosis), pustules, wilting, rot, hyperplasia (overgrowth) and hypertrophy, mold, deformation, discoloration, mummification, and tissue destruction. Various fungi, bacteria, viruses, and other pathogens spread these diseases (Nazarov et al., 2020).

The potato, scientifically known as *Solanum tuberosum* L., is the fourth most common crop produced worldwide after maize, wheat, and rice, and it is vital to the economy’s functioning. China, India, and Ukraine are the world’s top three potato exporters. Potatoes are susceptible to various diseases, the most common being early blight, late blight, black scurf, powdery scab, and soft rot (Liu et al., 2020). Most chemical substances are not used in disease control due to stringent regulations regularly managed and implemented globally. As a result, eco-friendly alternatives to traditional disease and pest management techniques are presently being researched and developed (Liu et al., 2020). Some of these techniques also use specific agricultural practices comparable to those used to cultivate products in a controlled setting. The agricultural technique of early harvesting, which entails collecting crops soon after the haulm’s destruction, is occasionally employed as a preventive strategy to curb the spread of black scurf and other potato diseases (Liu et al., 2020).

In addition, biocontrol has been enhanced by implementing “green harvesting” techniques, which involve the utilization of the hyper-parasite *Verticillium bigutatum* (Ebrahimi-Zarandi et al., 2021). *Trichoderma harzianum* has demonstrated efficacy as a biological control method for the pathogen *Rhizoctonia solani* in peas, radishes, and beans under controlled greenhouse conditions. Nonetheless, the predictability of its efficacy in practical scenarios posed a challenge (Ebrahimi-Zarandi et al., 2021). The addition of *T. harzianum* to the soil decreased the dormant inoculum of *Rhizoctonia solani*, reducing the quantity of *Rhizoctonia solani* that accumulated in both greenhouse and field settings. The application of *Laetisaria arvalis* onto potato seeds and soil modification using this fungus has been found to hinder the contamination of potato effectively stems, stolons, and sclerotial growth caused by *R. solani* (Ebrahimi-Zarandi et al., 2021). *Actinomycetes* are a group of unicellular, filamentous bacteria that exhibit branching morphology. They are renowned for their ability to produce antibacterial compounds (Vurukonda et al., 2018).

*Actinomycetes* are ubiquitous microorganisms that inhabit diverse ecological niches such as soil, air, plant debris, and sedimentary environments (Bhatti et al., 2017). The ability of the *Streptomyces* genus to produce a diverse array of secondary metabolites is a well-established characteristic within the *Actinomycetes* order. The secondary metabolites present antibacterial properties against diverse plant and human diseases (Viaene et al., 2016). Using antibiotic-producing bacteria as a biocontrol agent in the soil is a viable alternative to chemical antimicrobials. *Streptomyces* is known for its ability to synthesize a diverse array of bioactive organic compounds that elicit direct or indirect effects on plant growth and development and its antimicrobial properties (Viaene et al., 2016). For example, an organic compound produced by *Streptomyces yanglinensis* strain 3–10 can inhibit *A. flavus* from growing and producing toxins in stored soybeans (Gong et al., 2022). In naturally existing disease-suppressive soils, these bacteria hinder the growth of plant pathogens by generating secondary metabolites and engaging in resource competition, such as for carbon and iron (LeBlanc, 2022). *Streptomyces* species recently discovered are being seriously considered in the agricultural biocontrol field. *Streptomyces* is characterized by its capability to synthesize antimicrobial compounds (such as toxins, VOCs, and antibiotics) (Vurukonda et al., 2018). This enables the *Streptomyces* to help plants against pathogens that might otherwise damage them. Phytopathogen interactions may suppress innate plant responses to pathogens (Vurukonda et al., 2018). *Streptomyces* bacteria can further alleviate disease pressure by stimulating elements of the plant’s immune system and enhancing plant productivity (Olanrewaju and Babalola, 2019). In addition, it impacts soil fertility through its influence on different factors and its role as a nutrient enhancer (Vurukonda et al., 2018). Despite their proven ability to mitigate fungal plant pathogen-related diseases and their remarkable species diversity, there has been limited commercial development of *Streptomyces* strains as biological control agents (LeBlanc, 2022). Only Actinovate (containing *Streptomyces* lydicus WYEC108) and Mycostop (containing *Streptomyces* griseoviridis K61) are registered as commercial biopesticides for use on multiple continents. The limited availability of such products can be attributed, in part, to the difficulties in formulating them for commercial use and the disparities between results obtained in laboratory studies and those observed in field applications (Vurukonda et al., 2018).

## 2. Plant diseases

**FIGURE 1:**
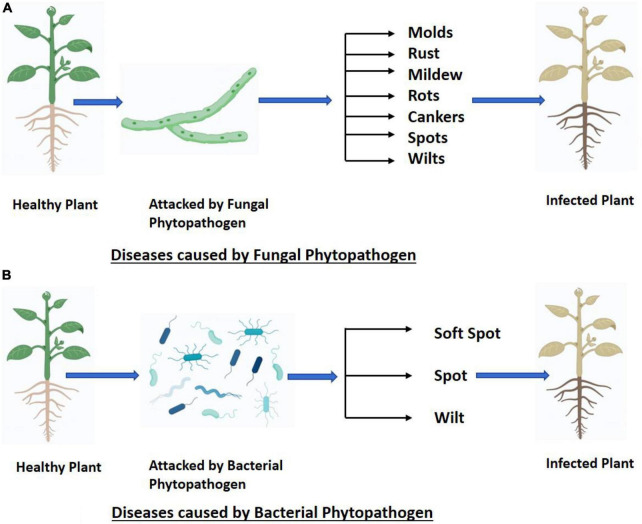
**(A,B)** Fungal and bacterial phytopathogens cause different types of plant diseases.

The broadening of international trade, climate change, and the limited availability of plant protection products have contributed to the development and appearance of new plant diseases, leading to substantial losses in agricultural production (Daranas et al., 2019). Understanding the factors that regulate disease emergence and spread has attracted remarkable attention from the world scientific community (Giraud et al., 2010). Plant diseases may express themselves in a variety of distinct manners depends upon type of pathogen i.e., fungal (Figure 1A) and bacterial (Figure 1B). The latest entrance of wheat blast-infected seed into Bangladesh is just one example of how pathogens can spread through damaged plant material, increasing the incidence, geographic range, or host range of the disease. The dispersal of pathogen spores over vast geographical regions can occur due to severe meteorological events like hurricanes (Ristaino et al., 2021).

### 2.1. Maize disease

Southern Mexican indigenous communities initially cultivated maize 10,000 years ago, and today, maize production surpasses that of wheat and rice in many countries. Maize serves various purposes, including human consumption, ethanol production, animal feed, corn starch, and corn syrup. It comes in six primary varieties: dent, pod, flint, popcorn, sweet corn, and flour. Sweet maize is grown for human consumption, while field maize is used for animal feed, maize oil production, whisky and bourbon distillation, and as a chemical feedstock (Chen et al., 2021). However, maize faces numerous threats, including fungal diseases, bacteria, viruses, nematodes, and parasitic plants. There are approximately 112 distinct diseases affecting maize crops, with around 70 transmitted through seeds (Xu et al., 2021). The most damaging seed-borne fungal diseases include *Gibberella* stalk rots (*Gibberella zeae*), *Fusarium verticillioides*, fake head smut (*Ustilaginoidea virens*), head smut, late wilt (*Harpophora maydis*), and black bundle (*Acremonium maydis*) (Lv et al., 2020). Other diseases include charcoal stalk rot, Anthracnose stalk rot (*Colletotrichum graminicola*), *Fusarium*, *Gibberella*, *Aspergillus* ear rots, charcoal ear rot (*Macrophomina phaseolina*), Corn smut (*Ustilago maydis*), *Penicillium*, common smut, downy mildew, black kernel rot, Horse’s tooth disease, *Alternaria* leaf blight, and *Acremonium zeae* stalk rot (Degani, 2021). Major seed-borne and seed-transmitted diseases of maize include black bundle, anthracnose rots, black kernel rot, crazy top downy mildew (*Sclerophthora macrospora*), brown stripe downy mildew, ear and root rot, Java downy mildew, horse’s tooth, Nigrospora ear rot, Philippine downy mildew disease, and Penicillium ear rot (Chen et al., 2021; Degani, 2021).

*Glomerella graminicola*, responsible for “Anthracnose Leaf Blight,” poses a threat to both wheat and maize crops and can also infect certain GMO cereals. This fungus causes anthracnose stalk rot worldwide, primarily damaging maize plants. The disease can manifest at any point during the growing season, leading to stem rot or leaf blight. Aspergillus flavus, a fungus with saprotrophic and pathogenic tendencies, is commonly found in various environments, especially cereal grains, tree nuts, and legumes (Chen et al., 2021). Post-harvest rot often occurs after harvesting or during storage and transport, with the name “flavus” referring to the yellow spore color. *Macrophomina phaseolina*, a Botryosphaeriaceae fungal plant disease, affects numerous plant species, including wheat, maize, peanuts, chickpeas, cabbage, tomatoes, soybeans, sweet onions, sunflowers, alfalfa, sesame, potatoes, and sorghum, causing damping-off, charcoal rot, seedling blight, and various stem and root rots (Degani, 2021; Degani and Dor, 2021). *Sclerophthora macrospora*, an Oomycota-pathogenic protist, infects maize, oats, rice, wheat, and turfgrass, leading to “crazy top” in maize and “yellow tuft” in turfgrass, particularly in Europe. Its lack of host specificity makes it a significant threat to various economically important crops. Gray Leaf Spot (GLS) is a major foliar fungal disease in maize caused by *Cercospora zeina* and *Cercospora zeae*, *Cercospora maydis*, with maize being its exclusive host. *Cercospora zeae maydis* populations are characterized by geographic distribution, growth rate, molecular analysis, and the presence of cercosporin toxins. Smut, or maize smut, is caused by the fungus *Ustilago maydis*, resulting in galls on the entire above-ground portion of the maize plant. Finally, southern maize leaf blight, caused by *Bipolaris maydis*, can be found wherever corn is grown worldwide (Yang et al., 2017). A list of major phytopathogens and corresponding disease caused in maize is listed in Table 1.

**TABLE 1:** List of major maize diseases, their causal agents, and yearly losses (Khokhar et al., 2014).

Sl. No.	Microbial pathogen	Disease name	% Losses annually
1.	*Erwinia carotovora* p var *zeae* / *Dickeya zeae*	Bacterial stalk rot	85
2.	*Fusarium graminearum*	Root rot	25–30
3.	*Harpophora maydis*	Late wilt	51
4.	*Macrophomina phaseolina*	Charcoal rot	25–32
5.	*Fusarium verticillioides*	Fusarium Stalk rot	10–42
6.	*Rhizoctonia solani f*. sp. *sasaki*	Banded leaf and sheath blight	0–60
7.	*Peronosclerospora*, *Sclerophthora*	Downy mildew	10–30
8.	*Fusarium verticillioides*	Ear rot	5–15
9.	*Ustilago zeae*	Common smut	40–100
10.	*Sporisorium reilianum*	Head smut	Up to 30
11.	*Alternaria tenuissima*	Alternaria leaf spot	3–7
12.	*Aureobasidium zeae*	Eye spot	14–44
13.	*Puccinia sorghi*	Common corn rust	18–49
14.	*Puccinia polysora*	Southern corn rust / **Polysora rust**	20–80
15.	*Cercospora zeae*	Gray leaf spot	5–30
16.	*Physoderma maydis*	Brown spot	6–20
17.	*Cochliobolus lunatus*	Curvularia leaf spot	10–60
18.	*Cochliobolus heterotrophic*	Southern corn leaf blight	15–46
19.	*Setosphaeria turcica, Exserohilum turcicum*	Northern corn leaf blight or **Turcicum leaf blight**	13–50
20.	Maize rough dwarf virus	Maize rough dwarf	10–70
21.	Maize dwarf mosaic virus	Maize dwarf mosaic	0–90

### 2.2. Wheat diseases

Wheat is the main source of calories and plant-derived protein in human food. According to the UN Food and Agricultural Organization, world wheat supply is sufficient. As the global population is expected to approach nine billion by 2050, production must expand (Figueroa et al., 2018). Wheat production is threatened by climate change, decreasing farmland, and unpredictable abiotic and biotic stresses. Diseases threaten world wheat supplies due to a perfect storm involving the advent of new pathogens and the reduction in wheat’s genetic variety brought on by the pursuit of elite high-performing cultivars. Pathogenic fungus limit wheat production. Rust infections have affected wheat production since its adoption. Wheat rust pathogens cause US$4.3–5.0 billion in annual losses worldwide (Juroszek and von Tiedemann, 2015). Rust species can infect diverse hosts, which is represented in formae speciales classification (ff. spp.). *Puccinia graminis* f. sp. *tritici* (Pgt), *Puccinia striiformis* f. *tritici* (Pst), and *Puccinia triticina* (Pt), are all members of the Basidiomycete family and the genus *Puccinia* (Singh et al., 2016). They are the pathogens that cause stem, stripe, and leaf rust, all diseases that affect wheat (Table 2).

**TABLE 2:** List of the most significant diseases of Wheat, their causative pathogens, and the disease’s primary symptoms (Figueroa et al., 2018; Waqar et al., 2018; Degani et al., 2021).

Microbial pathogen	Disease name	Primary symptoms of disease	Parts affected
*Blumeria graminis f*. sp. *tritici*	Powdery-mildew	The flowers, stem, sheath, and leaf have grayish/white powdery-growth. White blotches on leaves and stems indicate powdery-mildew. Powdery-growth turns black-dark and dries leaf and other parts.	Leaf, sheath, stem, and flowers
*Ustilago tritici*	Loose-smut	Wind-borne spores infect Loose Smut seeds during blossoming. Infected inflorescence grows from healthy-looking seeds. Infected heads appear earlier at this time. The entire flower often looks as black olive spores coated mass by a thin-gray coating, Powdery head after membrane rupture.	Seeds, leaf, sheath, stem, and flowers
*Puccinia triticina*	Brown-rust	Symptoms usually appear on upper-leaf blades, but awns, glumes, and sheaths can also be affected. Large numbers of orange to orange-brown Urediospores are housed within the pustules, which can be either round or somewhat oval in shape.	Leaf blades, sheaths, glumes, and awns
*Puccinia striiformis f*. sp. *tritici*	Stripe-rust or Yellow-rust	Mostly on leaves. Early crop leaves have bright yellow pustules (Uredia) in stripes. Yellow-orange stripes. Teliospores have lengthy stripes and are dull black. Leaves, necks, and glumes have pustules.	Leaf blades, sheaths, glumes, and awns
*Ustilago nuda ⋅ Puccinia graminis ⋅ Alternaria solani ⋅ Xanthomonas oryzae*	Black-rust	Most wheat plant aerial parts show symptoms, but lower and upper leaf surfaces, leaf sheaths, and stem are most common. Both sides of leaves, stems, and spikes have dark reddish brown pustules with masses of urediospores. Heavy infections coalesce pustules. Flecks may precede pustules. Infection sites feel scratchy before spore masses break through the epidermis. Surface tissues tear as spore masses burst through.	Leaf blades, sheaths, glumes, and awns
*Urocystis agropyri (G. Preuss) J. Schröt*	Flag-smut	From late seedling until maturity, stem, clum, and leaves show symptoms. Twisting and wilting leaves result from seedling infection. Leaf blade and sheath sori are grayish black. Black granular spores fill the sorus.	Stem, clum, leaves
*Tilletia tritici*	Hill-bunt or Stinking-smut	Systemic fungus affects 8–10-day-old seedlings and develops along shoot tips. During the flowering process, hyphae accumulates in the inflorescence and spikelets, transforming the ovary into a dark green smut sorus packed with chlamydospores. All spikelets on sick plants mature earlier.	*Plant*
*Tilletia indica Mitra*	Karnal-bunt	Due to the low occurrence of infected kernels on a head, Karnal bunt symptoms are hard to spot in the field. Sorus production spreads the glumes, although not as much as common bunt. Seed after harvest shows symptoms best.	*Seed, leaf, plant*
*Alternaria triticina, Bipolaris sorokiniana and Alternaria alternate*	Leaf-blight	Seedlings with vivid yellow margins have reddish brown oval markings. Multiple spots dry leaves in severe situations. *A. triticina*, *B. sorokiniana*, and *A*. *alternative* cause this complex disease.	Leaves
*Fusarium* spp.	Foot-rot	The disease mostly affects roots and seedlings, turning rootlets-brown. Stunted seedlings are pale-green. Fungal sporangia produce zoospores and oospores.	Roots
*Cochliobolus sativus, Pyrenophora tritici-repentis*	Helminthosporium leaf blotch	This condition causes dark brown, oval-shaped lesions. Lesions grow into light-brown to tan-centers with an uneven (21 on leaf; 22 on spike) dark-brown rims. Lower leaf infections start as chlorotic specks or patches. These infection spots grow, darken, and even merge. The disease can kill leaves or leaf sheaths.	*Leaf*, sheaths
*Rhizoctonia* spp., *Fusarium* spp., and *Pythium* spp.	Seedling-blight	Lesions caused by *Fusarium* typically begin in the leaf sheath at the base of the stem. This happens when crown roots split it as they emerge. This infection can move up the leaf sheath, causing long, dark brown streaks at the base of the stem. Ear-blight can cause yield loss and grain mycotoxin production.	Root, leaf, sheath, seed

Wheat stem (black) rust, caused by *Puccinia graminis* f. sp. *tritici* Ericks and Henn (Pgt), is widespread but less prevalent than the other two wheat rusts. In warm, damp climates, Pgt causes masses of red-brick urediniospores on vulnerable plants’ leaf sheaths, stems, glumes, and awns (Martinelli et al., 2015; Figueroa et al., 2018). Stem rust reduces grain size and plant lodging, reducing yield. Stem rust has been largely managed in many regions of the world, but forecasting models assuming no persistent resistance anticipate global losses of 6.2 million metric tons per year or greater under severe epidemics. *P. striiformis Westend*. f. sp. *tritici* (Pst), a pathogen common in temperate regions with cool and wet weather, causes wheat stripe (yellow) rust (Figueroa et al., 2018). In vulnerable cultivars, stripe rust causes 100% yield losses. Pst affects 88% of wheat cultivars worldwide, costing almost US$1 billion annually. Wheat stripe rust has been documented in over 60 countries and Pst has spread globally in the last 50 years. Pst populations in Europe, Australia, and North America are clonal, but some have high genotypic diversity (McIntosh et al., 2018). Western China and Central Asia have polymorphism populations, supporting the Himalayan and adjacent regions as the pathogen diversity hotspot where sexual recombination is widespread (Sharma et al., 2020). Leaf rust, the most widespread of the three wheat rust diseases, is caused by *Puccinia triticina Eriks*. Mild, damp climates support the pathogen. Kernel weight and grain per head decrease due to illness. Leaf rot causes temporal and regional variance in grain losses, yet it is economically significant. Leaf rust is an issue because the pathogen is diverse, produces new virulence profiles, and adapts to several conditions. The Ascomycete fungus *Zymoseptoria tritici*, *Parastagonospora nodorum*, and *Pyrenophora tritici-repentis* cause STB, SNB, and TS (Moolhuijzen et al., 2022). Blotch illnesses are these disorders. STB, the main leaf disease of temperate wheat, is caused by *Zymoseptoria tritici* (Zt). The Ascomycete fungus *Fusarium graminearum* causes *Fusarium* head blight (FHB), often known as wheat scab or ear blight, which prematurely ages wheat heads (Fg). Many regional species complexes of cereal-infecting *Fusarium* species create severe FHB epidemics. FHB is the worst wheat floral disease worldwide. Rain during crop anthesis makes wheat harvests susceptible to FHB (Xu et al., 2022). FHB disease reduces grain yield and quality, lowering harvest and marketability and accumulating sesquiterpenoid trichothecene mycotoxins like deoxynivalenol (DON) in the grain, posing a food safety and health risk to humans, animals, and ecosystems (Xu et al., 2022). The pathogenic fungus *Magnaporthe oryzae* Triticum pathotype (MoT) causes wheat blast (WB) (Paul et al., 2022). WB affects the head. Small elliptical lesions to bleaching and empty spikes are common signs. Yields have dropped 40–100%. MoT-caused foliar lesions have also been reported, but their impact on grain output is unknown. WB development requires 25°C and a 10-h soaking period (Ceresini et al., 2018).

### 2.3. Rice disease

Rice is grown on 161 million acres worldwide, producing 678.7 million tons of rice. Asia produces 612 million tons of rice on 143 million acres (Asibi et al., 2019). With a projected 34% increase in the world population to 9.3 billion by 2050, the goal of more production while losing less seems compelling due to the threat of pathogens and pest introductions due to increased human mobility, global trade, and climate change. Rice plants are infected by many devastating diseases like blast, leaf blights, sheath blight, sheath rot, brown spot, bakanae disease, etc., caused by fungi, bacteria, and viruses as mentioned in Table 3 (Singh et al., 2019; Abade et al., 2021; Ngalimat et al., 2021). These diseases reduce crop yield and quality. Pathogens cause a loss of 15–30% rice yield, costing 33 billion USD yearly. Management and prevention of pathogen-caused illnesses that impair rice yields are major challenges. Due to population booms, rice experts have struggled to find ways to produce nutritious food grains at cheaper costs. These must be done in the face of unforgiving plant diseases. Rice hosts 58 fungal (43 seedborne or seed-transmittable), 12 bacterial, 17 viral and mycoplasma-like, and over 30 nematode species (Chukwu et al., 2019; Ngalimat et al., 2021). Pathogens infect seeds, propagules, roots, nodes, panicles, and leaves. Pathogens may cause local or systemic infections that cause minor to severe crop loss. As rice is grown worldwide, so are its pathogens. Blast, brown spot, bacterial blight, sheath blight, and tungro are still inflicting harm, while new minor diseases, including bakanae, fake smut, grain discoloration, early seedling blight, narrow brown spot, and sheath rot have become important issues (Abade et al., 2021). In many rice-growing countries, *Helminthosporium oryzae*, *Rhizoctonia solani*, *Gerlachia*, *Pyricularia*, *Xanthomonas*, *Sclerotium*, and others have caused foliar diseases and stem, root, or leaf sheath issues (Chukwu et al., 2019). These diseases might cause 1–100% losses depending on growth conditions, varietal sensitivity, etc. Rice sheath rot is a complex disease caused by fungal and bacterial phytopathogens. *Sarocladium oryzae* and *Fusarium fujikuroi* species (*Fusarium fujikuroi*, *Fusarium verticillioides*, and other *Fusarium* spp.) complex are the main phytopathogens (Sankaran et al., 2010). *Pseudomonas fuscovaginae* is a bacterial pathogen (Liu et al., 2014). The main pathogen, *Sarocladium oryzae*, was first reported from Taiwan in 1922 as *Acrocylindrium oryzae*. *Sarocladium oryzae* was renamed in 1975 after the genus was formed. *Fusarium fujikuroi* (Nirenberg) causes foot rot, or bakanae, in rice. In recent years, north Indian basmati-growing regions have been plagued by the disease (Sunder et al., 2014; Kumar et al., 2016). Punjab, Haryana, eastern UP, Uttarakhand, and New Delhi, notably basmati-growing districts, are plagued by disease. Bakanae disease causes grain sterility, reducing yield. The illness can reduce field yield and quality by 70% (Zhu et al., 2022). *Ustilaginoidea virens* causes false or green smut, a rice disease in India. False smut disease of rice outbreaks began in Tamil Nadu, India, and spread worldwide (Prasher and Sharma, 2022). Smut balls replace one or more ripe plant kernels. Powdery dark green spores discharge as smut balls burst. *U. virens* thrives in 25–30°C and >90% relative humidity (Abade et al., 2021). Early seedling blight (*Sclerotium oryzae*, *Sclerotium rolfsii*) is a major developing disease in nursery beds during the cold season (winter months) (Gaire et al., 2023). Seedlings in the nursery bed begin withering. Later, fungal mycelia cover the seedlings. Fungal mats cover most roots and branches. Sclerotial bodies evolve into dark brown hard structures from white mustard-shaped ones (Chukwu et al., 2019; Abade et al., 2021). In Asia, high-yielding hybrid varieties have replaced traditional landraces, and chemical fertilizers and plant growth hormones have intensified crop production. Rice pathogens—the clever enemies—have become more relevant under these shifting settings. Management techniques had to include many formerly unimportant diseases. Many new pathotypes have emerged and some have disappeared (Parajuli et al., 2022). A list of major phytopathogens and related diseases caused in rice is listed in Table 3.

**TABLE 3:** List of the most significant diseases of rice, their causative pathogens, and the disease’s primary symptoms (Mehta et al., 2019; Singh et al., 2019; Abade et al., 2021; Ngalimat et al., 2021).

Microbial pathogen	Transmission type	Disease name	Average yield loss annually	Primary symptoms of disease	Parts affected
*Microdochium albescens*	Seeds, stubbles	Leaf-scald disease	15–20%	Dark-dark, oblong leaf tip lesions, translucent leaf tips, flower distortion, glume discoloration.	Grains, flower, leaf, coleoptiles
*Fusarium fujikuroi*	Water, Air, Seed	Bakanae disease	3.7–50%	Pale leaves, tall growth, reduced tillers, and full grains.	Grains, tillers, leaf, roots
*Rhizoctonia solani*	Water, Air	Sheath- blight disease	10–35%	Leaf lesions are round or ellipsoidal, greenish or gray to white with brown borders.	tillers, leaf
*Magnaporthe oryzae*	Air	Rice-blast disease (neck and node)	20–50%	Panicle cracking, banded infection on nodes, blackish or light brown lesions, grayish-brown neck lesions.	Neck, node, collar, leaf sheath, leaf, grains
*Sphaerulina oryzina*	Air	Narrow-brown spot disease	1–3%	Plant lodging, premature grain ripening, dark brown leaf lesions and a net blotch on leaf sheath	Glumes, panicle, leaf sheaths, leaf
*Bipolaris oryzae*	Air	Brown-spot disease	5–45%	Small, round or oval, dark brown to gray lesion with leaf borders of light reddish brown. Seedlings’ coleoptiles have small, round, yellow-brown blemishes.	Seeds, spikelet, glumes, sheath, leaf
*Ustilaginoidea virens*	Air	False-smut disease	35–45%	Grain with silky golden fruiting bodies.	Spikelet, grains
*Magnaporthe grisea / Pyricularia oryzae*	Air	Rice-blast (collar, panicle, and leaf)	30–75%	White to gray-green spindle-shaped patches with dark red, green, or necrotic margins	Neck, node, collar, leaf sheath, leaf, grains, seedling
*Xanthomonas oryzae pv. Oryzae*	Water, Air	Bacterial-blight disease	20–70%	Water-soaked or yellow-orange streaks, straw-colored leaves, seedling wilting	Seedling, leaf
*Pseudomonas fuscovaginae*	Seeds	Sheath-rown rot disease	72–98%	Discolored, malformed, or empty grains, irregular dark green, water-soaked lesions, yellow to brown leaf and seedling discolouration	Seedling, grains, sheath, leaf
*Sarocladium oryzae*	Air, infected parts of insects	Sheath-rot disease	3–20%	Sterile, dark brown rot panicles; empty, discolored seeds; irregular patches with dark reddish-brown edges	Grains, panicle
*Xanthomonas oryzae pv. oryzicola*	Stubbles, seeds	Bacterial leaf-streak disease	3–17%	Browning, drying, and water-soaked lesions in leaf veins	Leaf
*Xanthomonas rubrilineans*	Air	Red-stripe disease	2–5%	Necrotic, pin-sized, dark orange lesions on leaf and leaf sheath	leaf sheath, Leaf
*Nakataea oryzae*	Water, infected parts of insects	Stem-rot disease	30–80%	Black blemishes, chalky grains, unfilled panicles, lodging	Panicle, culms
*Rice tungro Bacilliform virus, Rice tungro spherical virus*	Green leaf-hopper	Tungro disease	20–95%	Leaf discoloration, stunting, decreased tillers, partially filled grains, stubble formation	grains, tiller, Leaf
*Rice yellow mottle virus*	Beetles, grass-hoppers, cows, mites, mechanical	Yellow-mottle disease	10–100%	Stunting, mottled, and twisted leaves, fewer tillers, discoloration, and poor panicle exsertion	grains, tiller, Leaf
*Rice ragged stunt virus*	Brown plant-hopper	ragged-stunt disease	35–80%	Yellow-brown, stunted leaves with serrated edges.	panicle, Leaf, grains
*Rice stripe virus*	Brown plant-hoppers	stripe- virus disease	30–100%	Stunting/mottling; chlorosis; yellowish-white stripes; necrotic streaks on leaves; folded, wilted, and droopy leaves; fewer tillers with many whitish to brown and malformed, premature panicles	Leaf, tiller
*Rice grassy stunt virus*	Brown plant-hopper	Grassy-stunt disease	10–35%	Plant stunting, grassy growth, rosette look with profuse tillering, short, narrow yellowish-green leaves with little red spots	Tillers, panicle
*Rice stripe virus*	Brown plant-hoppers	stripe -virus disease	30–100%	Stunting/mottling; chlorosis; yellowish-white stripes; necrotic streaks; folded, wilted, and droopy leaves; fewer tillers with many whitish to brown and premature panicles	Tillers, leaf

### 2.4. Potato diseases

The global potato production landscape is vast, with approximately 378 million tons of potatoes cultivated across roughly 19 million hectares of farmland. The majority of potato cultivation takes place in the northern hemisphere’s temperate regions due to the crop’s susceptibility to frost during the summer months. Potatoes exhibit adaptability to various environmental conditions, leading to significant production increases in several countries over the past two decades, particularly in emerging nations, with a notable surge in Asia and Africa Campos and Ortiz (2020). However, potato production faces numerous challenges, primarily from various diseases that affect both the tubers and the plants. More than 40 diseases can affect potatoes, caused by a range of microorganisms such as viruses, fungi, nematodes, insects, and bacteria. These diseases can lead to quality deterioration and yield losses of up to 22% in the potato production system, either directly or indirectly (Tiwari et al., 2020). One of the historically significant diseases is late blight, caused by the fungal pathogen *Phytophthora infestans*, which led to the devastating Irish potato famine in the 1840s. Late blight continues to pose a significant challenge to potato producers worldwide, with changing characteristics, including an increased affinity for tomato and potato plants (Kumbar et al., 2019). Other notable diseases mentioned include potato silver scurf caused by *Helminthosporium solani*, *Rhizoctonia solani* complex disease, and early blight caused by *Alternaria solani*. Each of these diseases can have a detrimental impact on potato production, leading to reduced yields and compromised tuber quality (Tomilova et al., 2020; Gorai et al., 2021; Tiwari et al., 2022).

## 3. Phytopathogenic microorganisms

Numerous phytopathogenic microorganisms generate active extracellular proteinases, which, in conjunction with other enzymes, serve a crucial function in their pathogenic processes. These enzymes include polygalacturonases, pectolyases, and xylanases, among others (Valueva and Mosolov, 2004).

Fungi are likely the most diverse group of environmentally and economically important organisms, particularly concerning their plant pathogenicity. Most fungal plant pathogens are classified within the phyla Basidiomycota and Ascomycota. Plant infections are categorized into various classes within the ascomycetes group, including Dothideomycetes (which encompasses *Cladosporium* spp.), Leotiomycetes (such as *Botrytis* spp.), and Sordariomycetes (including *Magnaporthe* spp.). The basidiomycetes, spread among the sub-phylum of *Ustilaginomycotina*, comprise two significant groups of plant pathogens: rusts (*Pucciniomycetes*) and smuts (Doehlemann et al., 2017). Crops are susceptible to fungal infections, either individually or in conjunction with other phytopathogens, throughout their growth cycle from seedling to seed maturation under natural conditions (Narayanasamy, 2011). Plant pathogenic fungi are responsible for causing various diseases in plants, including but not limited to Anthracnose, root rot, scab, canker, powdery mildew, dieback, leaf spot, blight, gall, damping off, rust, and wilt. These diseases are widely prevalent and can significantly impact plant health and productivity (Iqbal et al., 2018). Numerous phytopathogenic fungi induce crop diseases, leading to crop damage as mentioned in Table 4.

**TABLE 4:** Fungal phytopathogens with their respective host and disease causes (Narayanasamy, 2011; Doehlemann et al., 2017; Möller and Stukenbrock, 2017; Iqbal et al., 2018; Hariharan and Prasannath, 2021).

Name of fungus	Host	Disease	References
*Puccinia triticina Puccinia striiformis f*. sp. *tritici*	Wheat	Leaf rust	Möller and Stukenbrock, 2017
*Uromyces betae*	Sugar beet	Sugar beet rust	Möller and Stukenbrock, 2017
*Rhizoctonia solani*	Potato	Black scurf	Möller and Stukenbrock, 2017
*Phytophthora infestans*	Potato	Potato late blight	Möller and Stukenbrock, 2017
*Ustilago maydis*	Corn	Corn smut	Möller and Stukenbrock, 2017
*Fusarium fujikuroi*	Rice	Bakanae	Möller and Stukenbrock, 2017
*Colletotrichum falcatum*	Sugarcane	Red rot	Möller and Stukenbrock, 2017
*Alternaria* spp.	Pear	Black spot	Möller and Stukenbrock, 2017
*Phytophthora sojae*	Soybean	Phytophthora root rot	Möller and Stukenbrock, 2017
*Peronospora destructor*	Pear	Pear black spot	Möller and Stukenbrock, 2017
*Heterobasidion parviporum Heterobasidion annosum*	Conifers	Conifer root	Narayanasamy, 2011; Doehlemann et al., 2017; Iqbal et al., 2018; Hariharan and Prasannath, 2021
*Sporisorium scitamineum*	Maize	Smut	Narayanasamy, 2011; Doehlemann et al., 2017; Iqbal et al., 2018; Hariharan and Prasannath, 2021
*Sporisorium reilianum*	Sugarcane	Smut	Narayanasamy, 2011; Doehlemann et al., 2017; Iqbal et al., 2018; Hariharan and Prasannath, 2021
*Microbotryum silenes-dioicae*	White campion	Smut	Narayanasamy, 2011; Doehlemann et al., 2017; Iqbal et al., 2018; Hariharan and Prasannath, 2021
*Microbotryum lychnidis-dioicae*	Red campion	Smut	Narayanasamy, 2011; Doehlemann et al., 2017; Iqbal et al., 2018; Hariharan and Prasannath, 2021
*Melampsora larici-populina*	Poplar and larch	Rusts	Narayanasamy, 2011; Doehlemann et al., 2017; Iqbal et al., 2018; Hariharan and Prasannath, 2021
*Melampsora lini*	Flax	Rusts	Narayanasamy, 2011; Doehlemann et al., 2017; Iqbal et al., 2018; Hariharan and Prasannath, 2021
*Bipolaris sorokiniana*	Wheat, barley and grasses	Root diseases	Narayanasamy, 2011; Doehlemann et al., 2017; Iqbal et al., 2018; Hariharan and Prasannath, 2021
*Parastagonospora nodorum*	Wheat	Septoria nodorum blotch	Narayanasamy, 2011; Doehlemann et al., 2017; Iqbal et al., 2018; Hariharan and Prasannath, 2021
*Leptosphaeria maculans “brassicae” Leptosphaeria biglobosa “canadensis”*	Crucifers	Blackleg (Phoma stem canker)	Narayanasamy, 2011; Doehlemann et al., 2017; Iqbal et al., 2018; Hariharan and Prasannath, 2021
*Pseudocercospora musae Pseudocercospora fijiensis*	Banana	Black Sigatoka	Narayanasamy, 2011; Doehlemann et al., 2017; Iqbal et al., 2018; Hariharan and Prasannath, 2021
*Zymoseptoria tritici*	Wheat	Septoria tritici blotch	Narayanasamy, 2011; Doehlemann et al., 2017; Iqbal et al., 2018; Hariharan and Prasannath, 2021
*Zymoseptoria ardabiliae*	Wild grasses	Septoria blotch	Narayanasamy, 2011; Doehlemann et al., 2017; Iqbal et al., 2018; Hariharan and Prasannath, 2021
*Podosphaera plantaginis*	Plantains	Powdery mildew	Narayanasamy, 2011; Doehlemann et al., 2017; Iqbal et al., 2018; Hariharan and Prasannath, 2021
*Blumeria graminis f*. sp. *secalis*	Rye	Powdery mildew	Narayanasamy, 2011; Doehlemann et al., 2017; Iqbal et al., 2018; Hariharan and Prasannath, 2021
*Blumeria graminis f*. sp. *triticale*	Flax	Powdery mildew	Narayanasamy, 2011; Doehlemann et al., 2017; Iqbal et al., 2018; Hariharan and Prasannath, 2021
*Blumeria graminis f*. sp. *tritici*	Triticale	Powdery mildew	Narayanasamy, 2011; Doehlemann et al., 2017; Iqbal et al., 2018; Hariharan and Prasannath, 2021
*Blumeria graminis f*. sp. *hordei*	Wheat	Powdery mildew	Narayanasamy, 2011; Doehlemann et al., 2017; Iqbal et al., 2018; Hariharan and Prasannath, 2021
*Magnaporthe oryzae*	Barley	Blast disease	Narayanasamy, 2011; Doehlemann et al., 2017; Iqbal et al., 2018; Hariharan and Prasannath, 2021
*Pyricularia graminis-tritici*	Cereal crops and grasses	Persoonia	Narayanasamy, 2011; Doehlemann et al., 2017; Iqbal et al., 2018; Hariharan and Prasannath, 2021
*Ophiostoma ulmi*	Wheat	Dutch elm disease	Narayanasamy, 2011; Doehlemann et al., 2017; Iqbal et al., 2018; Hariharan and Prasannath, 2021
*Verticillium dahliae*	Elm Broad host range	Wilt diseases	Narayanasamy, 2011; Doehlemann et al., 2017; Iqbal et al., 2018; Hariharan and Prasannath, 2021
*Fusarium oxysporum f.* sp. *lycopersici*	Tomato	Wilt disease	Narayanasamy, 2011; Doehlemann et al., 2017; Iqbal et al., 2018; Hariharan and Prasannath, 2021
*Fusarium graminearum*	Cereal crops	Fusarium head blight	Narayanasamy, 2011; Doehlemann et al., 2017; Iqbal et al., 2018; Hariharan and Prasannath, 2021
*Nectria haematococca MPIV*	Broad host range	Fungal keratitis	Narayanasamy, 2011; Doehlemann et al., 2017; Iqbal et al., 2018; Hariharan and Prasannath, 2021

Bacterial infections have been observed to affect diverse plant species across the globe, causing various plant disease (Table 5). Phytopathogenic bacteria are a group of microorganisms that can inhabit the tissues or surfaces of plants and are of significant interest to the agriculture industry due to their impact on food-producing plants. Some of the symptoms produced by these organisms include cankers, spots, tissue rots, blights, and/or hormonal imbalances. Additionally, these organisms have the potential to induce excessive plant growth, promote root proliferation, trigger leaf epinasty, and interfere with growth (Kannan et al., 2015). Gram-negative bacteria constitute the majority (95%) of pathogenic bacteria, and gram-positive bacteria, which make up less than 5% of pathogenic bacteria, exhibit these differences (Liu et al., 2020). Bacterial infections substantially impact the sustainability of farming practices worldwide, potentially leading to negative consequences (Rubab et al., 2018). Several diseases are detected pre-harvest, and certain ones may continue to affect the quality of the product post-harvest.

**TABLE 5:** Bacterial plant pathogens with their host and disease (Kannan et al., 2015; Shafi et al., 2017; El-Sayed et al., 2019; Petersen et al., 2021).

Bacterial pathogen	Host	Disease	References
*Ralstonia solanacearum*	Tomato	Bacterial wilt	Kannan et al., 2015
*Pseudomonas syringae* pv. *porri*	Leek	Bacterial blight	Kannan et al., 2015
*Dickeya solani*	Potato	Soft rot/Blackleg	Kannan et al., 2015
*Xanthomonas campestris* pv. *vesicatoria*	Tomato	Bacterial spot	Kannan et al., 2015
*Xylella fastidiosa*	Grapevines	Pierce’s disease	Kannan et al., 2015
*Xanthomonas axonopodis* pv. *citrumelo*	Orange	Citrus bacterial spot	Kannan et al., 2015
*Streptomyces scabies*	Potato	Common scab	Kannan et al., 2015
*Pseudomonas tolaasii*	Mushrooms	Brown blotch disease	Kannan et al., 2015
*Pectobacterium carotovorum*	Lettuce	Soft rot	Kannan et al., 2015
*Xanthomonas oryzae*	Rice	Bacterial blight	Shafi et al., 2017
*Erwinia amylovora*	Apple	Fire blight	Shafi et al., 2017
*Erwinia carotovora*	Carrot	Soft rot	Shafi et al., 2017
*Xanthomonas citri*	Citrus	Citrus canker	Shafi et al., 2017
*Xanthomonas malvacearum*	Cotton	Angular leaf spot	Shafi et al., 2017
*Clavibacter michiganensis* subsp. *sepedonicus*	Potato	Ring spot	El-Sayed et al., 2019; Petersen et al., 2021
*Streptomyces scabies*	Potato	Scab	El-Sayed et al., 2019; Petersen et al., 2021

*Botrytis cinerea* is a fungal pathogen that is responsible for the development of gray mold disease. This pathogen can potentially cause significant damage to plant products and has been reported to infect more than 200 plant species (Buttimer et al., 2017). Other possibly harmful infections include *Colletotrichum musae*, which causes blossom end rot illnesses and Anthracnose in bananas, *Alternata alternata*, which causes alternaria rot in cherries; and *Penicillium expansum*, which causes blue mold in apples. Moreover, due to other novel attempts, many infections can be found before and after harvesting.

Viruses are non-cellular infectious agents with a unique ability to replicate only within living host cells. They can infect a wide range of organisms, including plants, animals, bacteria, and archaea. Viruses can either integrate into the host’s genome as inactive proviruses or actively replicate and manipulate the host’s biological processes. Suppression of viral gene transcription can lead to latent infections. Plants viruses primarily exist as single-stranded (ss) and double-stranded (ds) RNA viruses, as well as single-stranded and DNA-containing retroviruses. To infect plant cells, virions enter the cytoplasm through wounds created by mechanical damage to the cuticle and cell wall since they cannot pass through these barriers on their own. Once inside the cell, the virus un-coats, and DNA-containing viruses must penetrate the nucleus to initiate transcription and mRNA synthesis. Viruses encode at least two types of proteins: replication proteins required for nucleic acid synthesis and structural proteins forming the capsid. Some viruses also possess proteins responsible for virion movement between plant cells. Plant viruses can be transmitted both vertically (from parents to offspring) and horizontally (from diseased plants to healthy ones). They use small intercellular channels called plasmodesmata to penetrate neighboring cells, facilitating local infections. To infect an entire plant, a virus must enter the vascular system, moving passively through the phloem sieve tubes with the flow of substances, allowing it to infect cells distant from the primary site of infection. Some viruses are highly stable and resistant to heat, remaining viable in plant cells and derived products for extended periods. However, many plant viruses actively spread from infected plants to healthy ones through carrier organisms, divided into mechanical vectors (agents that do not propagate the virus) and biological vectors (where part of the viral life cycle occurs). Common vectors include arthropods, nematodes, and plant-feeding fungi.

Plant viruses pose a significant threat to various crops, with economic losses ranking second only to losses caused by other pathogens. They can infect numerous plant species, leading to potential crop losses of up to 98%, especially in subtropical and tropical regions. Notably, some infections may not exhibit obvious symptoms. Virus diseases in plants manifest in various ways, including growth suppression, discoloration (such as mosaic patterns and chlorosis), deformations (wrinkling, corrugation), necrosis, and impaired reproduction (flower sterility, parthenocarpy, shedding of flowers and ovaries).

Apart from viruses, viroids are another group of infectious agents, circular RNAs that cause diseases in plants and animals. They belong to the viral families Pospiviroidae and Avsunviroidae. Viroids lack a protein envelope (capsid) and consist of covalently linked ssRNA molecules, which are significantly shorter than viral genomes. Viroids cannot replicate autonomously and likely utilize host cell enzymes for replication through mechanisms such as rolling-circle replication. The exact molecular mechanisms of viroid pathogenicity are not fully understood but are believed to involve interactions with cellular kinases, gene expression alterations, protein induction, RNA interference, splicing disruption, and rRNA gene demethylation. Small changes in viroid nucleotide sequences can significantly affect their pathogenicity. Common symptoms of viroid diseases in plants include reduced growth, discoloration (chlorosis and anthocyanosis), and deformations in various plant organs.

Phytoplasmosis has a profound negative impact on both crop yield and quality. The extent of crop losses varies across different plants, with eggplants experiencing a 40% reduction, tomatoes suffering a 60% decrease, peppers facing a staggering 93% loss, potatoes encountering losses ranging from 30 to 80%, and cucumbers being particularly hard-hit with a 100% loss (Shafi et al., 2017). Plants affected by phytoplasmosis exhibit various disorders in their reproductive organs, including virescence, which involves the greening of flowers and the loss of normal pigmentation. Additionally, they may develop phyllodia, where parts of a flower transform into leaf-like structures, and proliferation, which leads to the emergence of multiple “pseudo” flowers instead of one. Furthermore, phytoplasmosis can result in symptoms like witches’ broom (excessive bushiness), dwarfism, wilting of plants, and leaf deformities. It’s worth noting that there is only one documented case of positive phytoplasmosis, which has a beneficial effect: phytoplasmosis in poinsettias, a popular seasonal ornamental plant, is known to have economically advantageous outcomes.

## 4. Antimicrobial biological compounds

Managing plant diseases caused by microorganisms is a crucial aspect of sustainable agriculture, which aims to produce healthy crops while minimizing environmental damage. Biological control is a promising approach that utilizes living organisms as natural enemies of plant pathogens (Bonaterra et al., 2022). The occurrence frequency of phytonematode genera revealed that Meloidogyne (35.41%), *Pratylenchus* (17.18%), *Tylenchorhynchus* (15.62%), and *Tylenchus* (11.45%) were the most prevalent genera among phytonematodes. Meloidogyne had the highest prominence value, followed by *Pratylenchus* and *Tylenchus*. The effects of animal manure treatments were sustained for an extended period in protective cucumber plants compared to the control treatment, resulting in the lowest population density of *Helicotylenchus* spp., *Tylenchorhynchus* spp., and *Pratylenchus* spp., as well as a significant reduction in galling and reproduction of Meloidogyne incognita in greenhouse experiment whereas in the field experiment, combinations of test manures and PGPR led to the highest improvement in tomato yield per plant compared to using animal manures alone. This coincided with reduced numbers of M. incognita populations and the maintenance of sustainable levels of beneficial nematodes (FLNs and PNs). Consequently, the application of a mixture of animal manures and PGPR emerges as a promising alternative to chemical pesticides for the biological control of nematode (Ali et al., 2022). Various *Streptomyces* strains underwent assessment for their potential to lower the population levels of the root-lesion nematode (RLN), *Pratylenchus penetrans*, within the roots of alfalfa (Medicago sativa) through experiments conducted in growth chambers. These same strains were previously observed to effectively control potato scab disease, which is caused by *Streptomyces* scabies, during field trials. Moreover, they exhibited the capability to restrain the *in vitro* proliferation of a diverse array of plant-pathogenic fungi and bacteria (Samac and Kinkel, 2001). An active compound possessing nematicidal properties was extracted from a strain identified as *Streptomyces* sp. 680560, and its structure was subsequently determined to be teleocidin B4. The nematicidal efficacy of this isolated compound, teleocidin B4, was subsequently verified (Kang et al., 2021). These actinobacteria typically inhabit the rhizosphere and rhizoplane, influencing the composition of the microbial community within the soil-root system. In some instances, they act as endophytes, forming a closer relationship with plant tissues. The mechanisms through which they promote plant growth include biofertilization and biostimulation effects, while their role in bioprotection relies on competitive mechanisms and the production of secondary metabolites. Among these metabolites, several compounds exhibit insecticidal properties, such as antimycin A, flavensomycin, macrotetralides, piericidins, and prasinons. *Streptomycetes* also produce highly effective and commercially successful metabolites, including avermectins, which are derivatives of macrocyclic lactones that impact the nervous system of insects. Avermectins interact with gamma-aminobutyric acid (GABA) receptors, initiating a series of events that lead to the inhibition of neurotransmission, resulting in neuromuscular paralysis and the eventual demise of the insects (Ruiu, 2020). Spectinabilin, a compound exhibiting nematicidal properties against both C. elegans and the southern root-knot nematode M. incognita, was isolated and characterized from *Streptomyces* sp. DT10. Spectinabilin demonstrated notable nematicidal activity against C. elegans L1 and L4 larvae, and it significantly impaired the mobility of C. elegans L4 larvae. Subsequent analysis indicated that spectinabilin operates on distinct targets compared to commonly used nematode treatments like abamectin and phosphine thiazole. These discoveries open up new possibilities for the development of more effective and environmentally sustainable strategies to combat parasitic nematodes while mitigating issues associated with resistance in these organisms (Sun et al., 2023). Meeting the growing demand for agricultural products requires optimizing production potential and minimizing crop losses attributed to common plant-parasitic nematodes. While chemical-based nematode management has proven effective in mitigating nematode-induced damage and yield reductions, the improper and irresponsible use of synthetic pesticides can have adverse effects on fauna, biodiversity, and natural predators and parasites. Farmers highly value biocontrol agents as a nematode management approach because it not only ensures safety but also reduces environmental pollution. There is a growing emphasis on the biological control of plant-parasitic nematodes through the utilization of plant growth-promoting rhizobacteria (PGPR) as biopesticides. Additionally, PGPR strains have the capability to enhance plant growth by producing a variety of secondary metabolites (Aioub et al., 2022). This method can be used alone or in conjunction with traditional pesticides, and it is more efficient and less harmful to the ecosystem. The use of biocides ranges from microbes and fungi to viruses and has gained attention from researchers worldwide due to their eco-friendly and effective properties. Agriculture is vital for sustaining the world’s population, and there is a growing need for increased crop production. However, the impact of weeds, plant diseases, and pests can cause significant damage to crops, leading to a decrease in yield and quality. The extensive use of chemical fertilizers and pesticides has resulted in environmental damage, the evolution of pesticide-resistant insects, and potential harm to human health. Therefore, alternative approaches, such as biological control, have been explored to minimize the impact of plant pathogens on crops (Keswani et al., 2019).

Fungi have gained increased attention as biocontrol agents due to their superior rates of sexual and asexual reproduction, rapid generation, and target-specificity (Thambugala et al., 2020). Although phytopathogenic fungi are a common source of plant disease, many fungal species have developed defense mechanisms to repel these invaders. Only a few species of *Trichoderma* are cited as biological control agents due to their strong opportunistic traits (Woo et al., 2014). *Trichoderma* spp. has a diverse metabolism and can use many substrates, producing hundreds of chemicals dispersed in more than 120 secondary metabolite structures (Malmierca et al., 2016). Plant growth-promoting bacteria (PGPB) is a type of bacteria that supports plant growth (Morales-Cedeño et al., 2021). Bacterial 1-aminocyclopropane-1-carboxylic acid (ACC) deaminase converts the ACC molecule to ammonia and -ketobutyrate under stress or pathogen assault, preventing the production of ethylene and promoting plant growth and longevity (Orozco-Mosqueda et al., 2020). Recently, there has been a surge in identifying and characterizing *Bacilli* strains with enhanced resistance to salty conditions, promotes numerous plant specie’s growth. *Bacilli* interact with plants in various beneficial ways (Tahir et al., 2019).

### 4.1. *Streptomyces*

*Actinomycetes* are distributed in various natural ecosystems such as rhizosphere soil, agricultural soil, marine and freshwater habitats, limestone, sponges, volcanic cave, desert, insect gut, goat feces, and endophytic plants, as reported by Selim et al. (2021). These microorganisms can produce secondary metabolites that are not directly linked to their growth, maintenance, or reproduction, making them unique due to their broad metabolic range and potential for generating novel compounds (Elmallah et al., 2020). Marine *Actinomycetes*, in particular, have been found to produce a diverse array of antibiotics, accounting for over 45% of ecologically significant bioactive metabolites (Jakubiec-krzesniak et al., 2018).

More than 700 species constitute the family Streptomycetaceae (order *Actinomycetales*), of which the genus *Streptomyces* is the most prominent. Their DNA has a G + C content greater than 70% (Law et al., 2017; Olanrewaju and Babalola, 2019; Pacios-Michelena et al., 2021) and they are Gram-positive, neutrophilic, facultatively aerobic, mesophilic filamentous bacteria with a growth temperature between 25 and 35°C (Law et al., 2017). Biosynthetic gene clusters (BGC) in *Streptomyces* bacteria code for the enzymes needed to produce the bacterium’s secondary metabolites (SMs). In most cases, they are governed by complicated and stringent transcriptional regulation. Various nutritional and environmental factors activate distinct regulatory mechanisms that control *Streptomyces* SM production. Most *Streptomyces* are successful colonizers of the rhizosphere. Endophytes are a subset that lives within the host plant and colonizes its tissues (Trejo-Estrada et al., 1998; Muangham et al., 2015; Thambugala et al., 2020). The production of cellulases, chitinases, lipases, and beta-1,3-glucanases, as well as the synthesis of siderophores, phytohormones, or amino acids, may be responsible for these traits. There are three basic strategies by which *Streptomyces* can exert its antagonistic actions against pathogens: compete for space and nutrients, antibiosis, and parasitism (Sadeghi et al., 2017).

### 4.2. Space and nutrients

Certain *Streptomyces* strains demonstrate antagonistic and antimicrobial capabilities against pathogens found in aquaculture environments. They achieve this by generating inhibitory substances like bacteriocins, siderophores, hydrogen peroxide, and organic acids, which are used to vie for nutrients and attachment sites within the host, thereby inhibiting the growth of these pathogens (Butt et al., 2023).

### 4.3. Antibiosis

*Streptomyces* many secondary metabolites which adversely affect the growth of pathogens, resulting in antibiosis (de Lima Procópio et al., 2012).

### 4.4. Parasitism

The main genus of *Actinomycetes* is *Streptomyces*. Through competition or parasitism, its member species manage plant parasitic nematodes (Jin et al., 2019).

**FIGURE 2:**
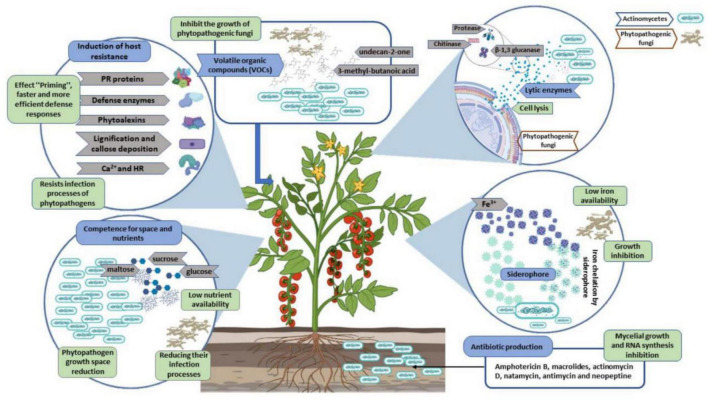
Principal phytopathogenic fungus antagonistic mechanisms of actinomycete/*Streptomyces*.

**FIGURE 3:**
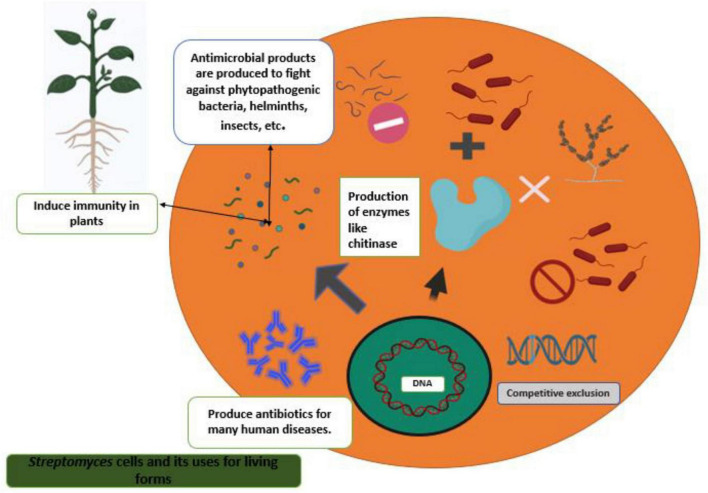
*Streptomyces* strains as a potential biocontrol agent against various pathogenic organisms and their mechanisms.

Plant root surfaces are a prime colonization site for *Streptomyces* spp., which can also thrive in a wide range of soil types and release spores to ensure their survival in harsh environments (Law et al., 2017). *Streptomyces* strains produce a wide array of antibiotics, volatile organic compounds (VOC), which are effective against diseases and disrupt bacterial cell-cell communication (quorum sensing), as well as a number of enzymes that destroy the cell wall of fungi as shown in Figures 2, 3. During their metabolic activities, *Streptomycetes* produce a number of lytic enzymes. They significantly contribute to carbon recycling by decomposing various biopolymers derived from dead plants and animal debris. These organic molecules are broken down by exoenzymes; they include xylan, chitin, and cellulose. Carbohydrate importers, which typically recognize mono- and disaccharides, direct the products into the cell. Bentley et al. (2002) sequenced all the genomes of the model organism *Streptomyces coelicolor* A3 and found 172 genes encoding secreted proteins like hydrolases, chitinases, cellulases, lipases, nucleases, and proteases, as well as 81 ATP-binding cassette (ABC) permeases that may be used to uptake sugars, oligopeptides, nucleosides, and drugs. *Streptomycetes* have a stronger metabolic capacity than other bacteria by a factor of 5–10, as evidenced by their larger levels of exoenzymes and ABC systems (Vurukonda et al., 2018). *Streptomyces* strains can be cultivated and nourished in a variety of ways to considerably alter their ability to produce antimicrobials. Lack of nutrients can affect hyphae formation, which in turn leads to the production of these antimicrobial chemicals. Cells perceive these environmental signals as stress, and thus, these compounds are categorized as stress metabolites owing to their function in enabling adaptability. In addition, *Streptomyces* antibiotic regulatory proteins and lysosomal acid lipase are two examples of species-specific enzymes that play a vital role in controlling metabolic pathways. The metabolites produced by *Streptomyces* are dependent on the nature of the signals it receives and sends. Invasion by pathogens triggers a signal, food deficiency triggers a signal, etc. Consequently, the ability of the producer organism and the composition of the culture medium are crucial factors in the production of secondary metabolites (Jakubiec-krzesniak et al., 2018; Keswani et al., 2019). Fermentation can be a key step in the production of secondary metabolites. Nutrients (nitrogen, phosphorus source, and carbon), growth rate, enzyme inactivation, and some variable factors (oxygen supply, temperature, light, and pH) are the primary factors that can be modified. Additionally, *Actinomycetes*’ strain-specific synthesis of important metabolites varies qualitatively and quantitatively (Muangham et al., 2015).

Furthermore, the diverse metabolic capabilities of the organism have facilitated their ability to inhabit various ecological niches and utilize a wide range of carbon and nitrogen substrates. While the pH range of the genus is typically between 6.5 and 8, it has been observed that specific strains can thrive in environments with a pH of 9 or greater. *Streptomyces* can produce mycelia and spores, which serve as a mechanism of spreading and resistance, facilitating survival during prolonged periods of water scarcity and nutrient deprivation (Pacios-Michelena et al., 2021). The genome of *Streptomyces* harbors over 20 gene clusters that synthesize secondary metabolites of notable antimicrobial significance, that exhibit the potential to address antimicrobial resistance (Hopwood, 2019). *Streptomyces* is characterized by its DNA-DNA hybridization and 16S rDNA analysis in contrast to other actinobacteria (Kumar et al., 2012). *Actinomycetes* isolated from hypersaline soils, such as *Streptomyces alboflavus*, *Micromonospora* species, *Nocardia* species, and *Streptomyces griseoflavus*, have also demonstrated antifungal activity against various fungal species, including *Aspergillus niger*, *Fusarium* species, and *Cryptococcus* species (Karuppiah and Mustaffa, 2013). Hypersaline soils are characterized by a *salt* content ranging from 9 to 23% and are found in various habitats such as saline soils, saline lakes, and salterns.

## 5. Antimicrobial compounds produced by *Streptomyces* spp.

Recent studies have emphasized using *Streptomyces* species as a biocontrol agent to counteract bacterial and fungi diseases that can affect plants (Suárez-Moreno et al., 2019). Multiple studies have shown that *Streptomycetes* exhibit efficacy against many phytopathogenic fungi, such as *Magnaporthe oryzae*, *Pyrrhoderma noxium*, *Phytophthora capsici*, *Rhizoctonia solani*, *Puccinia triticina, Pythium aphanidermatum*, *Fusarium verticillioides*, *Botrytis cinerea*, and *Pythium ultimum* (Panchalingam et al., 2022). Moreover, as numerous investigations indicate, *Streptomyces* exhibits significant potential for synthesizing secondary metabolites, including antibiotics, growth promoters, and herbicides (Harir et al., 2018). The genus, as mentioned above, is accountable for synthesizing 70% of the antibiotics currently utilized for therapeutic purposes in the treatment of diverse human diseases and constitutes 66.67% of all antibiotics that occur naturally (Sivalingam et al., 2019). The quantity of antimicrobial compounds produced by *Streptomyces* strains is contingent upon nutritional and environmental factors. The development of fungal hyphae and the production of antimicrobial compounds may be impeded in instances of nutrient deficiency. These compounds are generally identified as stress metabolites due to their adaptation function. The production of antibiotics is facilitated by different species and strains of *Streptomyces*, as presented in Table 6. The control of metabolic pathways is contingent upon the existence of various enzymes that are limited to specific organisms. These enzymes include lysosomal-acid lipase and the *Streptomyces* bacteria’s regulating proteins for drugs.

**TABLE 6:** Antibiotics produced by *Streptomyces species* along with their target pathogens and applications.

*Streptomyces* sp.	Trade name of the antibiotic	Target pathogen	Application	Country	References
*Streptomyces lydicus*	Actinovate	*Septoria glycines*	Prevent the leaf and root from fungus attack.	Guyana	Himmelstein et al., 2014
*Streptomyces griseoviridis strain K61*	Mycostop	*F. oxysporum, F. solani*	Control or inhibit many wilt and root rot pathogenic fungi.	Italy	Minuto et al., 2006
*Streptomyces* strains YCED9	Nigericin	*R. solani-P and S. homeocarpa*	Suppresses turfgrass diseases.	Moscow	Trejo-Estrada et al., 1998
*Streptomyces* sp. JCK-6131	Buramycin	*Bacterium ralstonia (Pseudomonas solanacearum)*	Suppresses the bacterial wilt of tomato.	Republic of Korea	Le et al., 2021
*Streptomyces flaveus* A11	Manumycin	*Alternaria, Magnaporthe grisea, Cladosporium cucumerinum and Phytophthora capsici*	Used against the diseases caused by given fungal phytopathogens.	Republic of Korea	Hwang et al., 1996
*Streptomyces hygroscopicus*	Rapamycin	*Verticillium dahliae*	Immunosuppressive, Antifungal	Republic of Korea	Kim et al., 2014
*Streptomyces* sp.	Anisomycin	*Erysiphe polygoni*	Suppresses the growth of annual grassy weeds like common crabgrass, bean mildew.	United States	Schumacher and Hall, 1982
*Streptomyces hygroscopicus*	Carbocyclic confomycin and hydantocidin	*Phytophthora*	Controls many weed	Japan	Nakajima et al., 1991
*Streptomyces griseus*	Faerifungin	*Fusarium oxysporum*	Inhibit Asparagus root diseases.	Japan	Kobinata et al., 1993
Streptomyces padanus	Fungichromin	*R. solani, Aphanomyces cochlioides, Fusarium, Alternaria*	Work on damping off of cabbage.	Taiwan	Huang et al., 2007
*Streptomyces violaceusniger*	Tubercidin	*Phytophthora capsici*	Used against phytophthora blight of pepper.	Republic of Korea	Hwang et al., 1994
*Streptomyces kasugaensis*	Kasugamycin	*Magnaporthe grisea*	Used against rice blast disease.	Japan	Kasuga et al., 2017
*Streptomyces melanosporofaciens* EF-76 AND EF-54	Geldanamycin	*Sarcoptes scabies*	Used against potato scab.	Canada	Díaz-Cruz et al., 2022
*Streptomyces hygroscopicus*	Gopalamycin	*Puccinia triticina*	Used against brown rust of wheat.	Indonesia	Sheng et al., 2015
*Streptomyces hygroscopicus*	Geldanamycin	*Rhizoctonia*	Used against pea root rot disease.	Moscow	Yuan and Crawford, 1995
*Streptomyces malaysiensis*	Malayamycin	*Mycosphaerella graminicola, Phytophthora capsici*	Used for wheat blotch.	United Kingdom	Li et al., 2008
*Streptomyces* sp. KNF 2047	Neopeptine A and B	*Podosphaera xanthii* and *Erysiphe cichoracearum*	Used against *Phytophthora* blight of pepper.	Japan	Satomi et al., 1982
*Streptoverticillium rimofaciens*	Mildiomycin	*Erysiphe, Microsphaera, Oidium, Leveillula*, and *Sphaerotheca*	Inhibit powdery mildew.	Japan	Kishimoto et al., 1996
*Streptomyces griseochromogenes*	Blasticidin S	*Magnaporthe grisea*	Covers broad ranges of plant diseases.	Japan	Kishimoto et al., 1996
*Streptomyces hygroscopicus* 5008	Validamycin/jinggangmycin	Various fungal and bacterial spp.	Covers broad ranges of plant diseases	Japan	Zhang et al., 2018

The metabolites synthesized by *Streptomyces* depend on the signals the organism receives and transmits. An example of this is the activation of a signal in response to the presence of a pathogen or a deficiency in nutrients. Butyrolactone is a key signaling molecule that promotes intercellular communication among PSPG (Plant Secondary Product Glycosyltransferases) cells via direct cell-to-cell contact (Olanrewaju and Babalola, 2019). The *Streptomyces* strain C, which produces siderophores, has been found to enhance the growth of wheat and its iron uptake capacity under saltwater conditions (Sadeghi et al., 2017). Siderophores legonoxamine A and B, members of the hydroxamate family, are produced by the strain of *Streptomyces* known as MA37 (Maglangit et al., 2019). Four strains of *Streptomyces*, namely *S. violaceusniger* YCED9, *S. lydicus* WYEC108, *S. saraceticus* KH400, and *S. griseoviridis* K61, are represented by six distinct marketable combinations. These strains are used to biocontrol bacterial and fungal diseases associated with the soil (Palaniyandi et al., 2013).

## 6. Use of antimicrobial compounds produced by *Streptomyces* in the agriculture

The agricultural sector commonly engages in the inappropriate disposal of pesticides, resulting in the chemical pollution of the nearby environment’s soil, air, and water. Pesticides that pose a significant risk to human wellbeing are readily available and can be obtained through authorized and unauthorized means, presenting a global concern. The detection of 10.5 metric tons of banned pesticides and/or insecticides with fraudulent labeling being imported into the European Union from China underscores the prevalence of uncontrolled and illicit pesticide application. The circumstance mentioned above presents a noteworthy peril to the wellbeing of the general populace, given that individuals could potentially encounter perilous concentrations of said chemicals, thereby leading to the grave and extensive health ramifications (Abraham and Gajendiran, 2019). Various types of diseases caused by bacteria, viruses, fungi, and nematodes substantially impact agricultural productivity on a global scale. The decrease in global food production caused by plant diseases was attributed to fungal and bacterial pathogens, which accounted for 42 and 27% of the decline, respectively. The potential exacerbation of this trend may be attributed to the impact of climate change. The utilization of biological control has experienced a notable increase due to its various advantages, including minimal residual toxicity, reduced environmental contamination, and more excellent pest resistance compared to chemical control (Le et al., 2022). The abundance of agro-active substances and biological control compounds found in *Actinomycetes* has captured the interest of researchers in diverse agricultural domains, owing to their considerable potential for practical utilization (Tables 7, 8). The *Streptomyces* genus has garnered considerable significance in the pharmaceutical sector owing to its capacity to synthesize one or more categories of antibiotics in nearly 75% of its constituent species.

**TABLE 7:** *Streptomyces* antimicrobials in the control of plant fungal pathogens.

*Streptomyces* species	Fungal pathogen	Crop	Disease	Antimicrobial metabolites	Country	References
*Streptomyces albulus* NJZJSA2	*F. cucumerinum, Sclerotinia sclerotiorum*	Cucumber, Oilseed rape	Fusarium wilt of cucumber. Sclerotinia stem rot of oilseed.	4-methoxystyrene.	Thailand	Wu et al., 2015
*Streptomyces setonii* WY228	*Ceratocystis fimbriata*	Sweet potato	Black spot disease.	2-Ethyl-5-methylpyrazine and dimethyl disulfide.	China	Gong et al., 2022
*Streptomyces angustmyceticus*	*Colletotrichum* sp. and *Curvularia lunata*	Cabbage	Leaf spot of Tokyo Bekana cabbage.	β-1, 3-glucanase.	Tokyo	Wonglom et al., 2019
*Streptomyces* strain CACIS-1.5CA	*Colletotrichum musae, Alternaria* sp., *Rhizoctonia* sp., *Colletotrichum* sp. M1.2	Banana, Tomato, Papaya, Mango, Grapes, Pepper	Anthracnosis, Black spot, Rot mango fruit, Soft rot.	Polyketide synthase (PKS) type.	Mexico	Evangelista-Martínez, 2014
*Streptomyces humidus*	*Phytophthora capsici*	Pepper	*Phytophthora* blight of pepper.	Phenylacetic acid.	Republic of Korea	Hwang et al., 2001
*Streptomyces cacaoi* vor. *Ascensus*	*Rhizoctonia solani* Kuhn.	Rice	Rice, Sheath blight of rice.	Polyoxin A and D.	Germany	Harir et al., 2018
*Streptomyces* sp. AB-88M	*P. oryzae, B. cinerea*	Wheat, Grapes	Wheat blast, gray mold disease.	AC-1	Japan	Matsuyama, 1991
*Streptomyces rimosus*	*Pythium* spp.	Safflower	Damping-off of safflower	RhizovitR.	Croatia	Leščić et al., 2001
*Streptomyces koyangensis* strain VK-A60.	*Colletotrichum orbiculare*	Watermelon	Infection of watermelon.	4 Phenyl 3 butenoic acid.	Republic of Korea	Lee et al., 2005
*Streptomyces psammoticus* KP1404	*Aspergillus oryzae*	Rice	Black rice bran.	Strevertenes.	Republic of Korea	Kim et al., 2011
*Streptomyces griseus H7602*	*P. capsici*	Tomato	Tomato root and crown rot.	1 H-Pyrrole-2-Carboxylic acid (PCA)	Republic of Korea	Nguyen et al., 2015
*Streptomyces griseorubens* E44G	*F. oxysporum f.* sp. *lycopersici*	Tomato	*Fusarium* wilt of tomato.	F31 D-CF	Saudi Arabia	Rashad et al., 2017

**TABLE 8:** *Streptomyces* antimicrobials in the control of plant bacterial pathogens.

*Streptomyces* species	Bacterial pathogen	Crop	Disease	Antimicrobial metabolites	References
*Streptomyces* sp. strain J145	*Xanthomonas campestris pv. campestris*	Cabbage	Black rot	α-1-Sorbofuranose and β-D-altrofuranose	Solanki et al., 2016
*Streptomyces termitum* ATC-2	*Xanthomonas oryzae pv. oryzae*	Rice	Bacterial blight in rice	Aloesaponarin II	Solanki et al., 2016
*Streptomyces humidus*	*Pseudomonas syringae pv. syringae*	Almond	Bacterial canker	Phenylacetic acid and sodium phenylacetate	Solanki et al., 2016
*Streptomyces diastatochromogenes*	*Erwinia carotovora*	Potato, Carrot, and Cabbage	Bacterial soft rot	Oligomycin	Doolotkeldieva et al., 2016
*Streptomyces hydrogenans* IB310	*Agrobacterium tumefaciens, Pseudomonas syringae, Xanthomonas campestris*	Cabbage, Almond	Soft rot, black rot and crown gall disease	Actinomycin D	Kulkarni et al., 2017
*Streptomyces* strain 22-4	*Xanthomonas axonopodis, Ralstonia solanacearum, Calvibacter michiganensis*	Tomato	Tomato bacterial wilt	Cycle (I-Pro-Tyr) and Cycle (α-Pro-I-Tyr)	Wattana-Amorn et al., 2016
*Streptomyces* sp. PNM-9	*B. glumae and B. gladioli*	Rice	Bacterial panicle blight disease	Two methyl-N-(2-phenylethyl)-butanamide and 3-methyl-N-(2-phenylethyl)- butamide	Betancur et al., 2019
*Streptomyces* strain FJAT-31547	*Ralstonia solanacearum*	Tomato	Bacterial wilt of tomato	n-hexadecanoic acid	Zheng et al., 2019
*Streptomyces* strain JJ45	*Xanthomonas campestris pv. campestris*	Cabbage	Black rot	α-L-sorbofuranose (3-2)- β- D-altrofuranose	Muangham et al., 2015
*Streptomyces* sp. AN090126	*Ralstonia solanacearum, Xanthomonas euvesicatoria, Sclerotinia homeocarpa*	Tomato, Red pepper	Tomato bacterial wilt, Red pepper leaf spot, creeping bentgrass dollar spot	Dimethyl sulfide and trimethyl sulfide	Le et al., 2022
*Streptomyces* sp. JCK-6131	*Ralstonia solanacearum*	Apple, tomato	Apple juice blight, tomato bacterial wilt	Streptothricin E acid, Streptothricin D and 12-Carbamoyl streptothricin D	Le et al., 2021
*Streptomyces* sp. 161a	*Burkholderia glumae and Burkholderia gladioli*	Rice	Bacterial panicle blight disease	Cyclo-tetrapeptides and diketopiperazines	Betancur et al., 2019

Furthermore, this particular genus has significantly contributed to the advancement of approximately sixty percent of novel insecticides and herbicides within the past 30 years. A significant amount of research is being conducted worldwide to develop efficacious compositions that incorporate *Actinomycetes* as a bioactive component (Le et al., 2022). *Streptomyces* is a noteworthy origin of biologically active substances, including vitamins, plant growth hormones, alkaloids, enzymes, and enzyme inhibitors. According to Shojaee et al. (2014), the biological stabilization of soils and the promotion of crop productivity are facilitated by soil *Streptomycetes*, which aid in the decomposition of organic materials.

The genus *Streptomyces*, which is included in the phylum Actinobacteria, exhibits a wide variety of processes, including the production of antibiotics, the degrading of fungal cell walls, engaging in competitive interactions, and hyper-parasitism. The effectiveness of these mechanisms of operation has been exhibited as biological control compounds, either autonomously or in combination with other biological control compounds. Numerous *Streptomyces* species, including *S. lividans*, *S. plicatus*, *S. humidus*, *S. scabies*, *S. violaceusniger*, *S. aureofaciens*, *S. hygroscopicus*, *S. olivaceoviridis*, *S. lydicus*, *S. avermitilis*, and *S. roseflavus*, have been identified for their capacity to produce highly effective bioactive substances that demonstrate effectiveness against diverse pathogenic fungi. Additionally, certain strains of this genus have been carefully chosen for their effectiveness as biological agents in managing diverse plant diseases (Kanini et al., 2013). The efficacy of products obtained from diverse *Streptomyces* species has been successfully demonstrated through production and experimentation, specifically in managing select plant diseases. One instance of pathogen inhibition entails the utilization of *Streptomyces griseoviridis* strain K61 to inhibit *Ceratocystis radicicola*, the etiological agent accountable for inducing black burns on date palms (MycostopR^®^). In addition, the bacterial strain *S. lydicus* WYEC108 was utilized to produce Micro108^®^ and Actinovate^®^ commercially used biocontrol products. Similarly, the strain *S. saraceticus* KH400, in combination with iron, was employed in the manufacturing process of YAN TEN^®^ biocontrol product (Bubici, 2018).

## 7. Challenges associated with the use of *Streptomyces* in the control of plant diseases

*Streptomyces* hold promise in agriculture, however, there are several challenges associated with its use in controlling plant diseases that need to be addressed for its effective deployment (Solanki et al., 2016; Le et al., 2021).

### 7.1. Specificity

*Streptomyces* strains vary widely in their effectiveness against different plant pathogens. Finding the right strain that targets a particular disease without harming beneficial microorganisms can be challenging. Ensuring specificity is crucial to prevent unintended ecological disruptions in the soil (Kasuga et al., 2017).

### 7.2. Environmental factors

*Streptomyces* performance can be greatly influenced by environmental factors such as temperature, humidity, and soil pH. Maintaining optimal conditions for its growth and activity can be demanding, limiting its effectiveness in diverse agricultural settings (Le et al., 2021).

### 7.3. Competition with native microflora

When introduced into the soil, *Streptomyces* must compete with the native microbial community for resources and niche space. This competition can reduce the survival and establishment of *Streptomyces*, undermining its ability to suppress pathogens effectively (Nguyen et al., 2015).

### 7.4. Persistence

*Streptomyces* can have limited persistence in the soil. Its beneficial effects may diminish over time, necessitating repeated applications, which can be costly and labor-intensive for farmers.

### 7.5. Resistance development

Just as with chemical pesticides, there is a risk of pathogens developing resistance to *Streptomyces*-based biocontrol agents. The rapid evolution of resistance can render these agents ineffective over time, necessitating the development of new strains or strategies (Muangham et al., 2015).

### 7.6. Regulatory hurdles

Regulatory approval for the use of *Streptomyces*-based biocontrol agents can be a lengthy and costly process. Ensuring that these agents meet safety and efficacy standards is essential but can impede their widespread adoption (Sun et al., 2023).

### 7.7. Production and formulation

Mass production and formulation of *Streptomyces*-based biocontrol agents can be complex and expensive. Achieving consistency in terms of viable spore count and product quality is crucial for their commercial viability (Sun et al., 2023).

### 7.8. Integration with conventional practices

Incorporating *Streptomyces*-based biocontrol into existing agricultural practices can be challenging. Farmers may need education and support to understand how to effectively integrate these agents with their current pest management strategies.

### 7.9. Variable results

*Streptomyces*’ effectiveness can vary from season to season and between different crop types. Predicting its performance accurately can be difficult, making it less reliable than some chemical alternatives (Zheng et al., 2019).

### 7.10. Consumer acceptance

There can be resistance from consumers who are unfamiliar with or skeptical of biological control methods. Public perception and acceptance are essential factors in the adoption of *Streptomyces*-based solutions (Wu et al., 2015).

In conclusion, while *Streptomyces* shows promise as a biocontrol agent for plant diseases, it is not without its challenges. Addressing these challenges requires ongoing research and development efforts, as well as collaboration between scientists, regulators, and farmers. By overcoming these obstacles, *Streptomyces*-based biocontrol agents could play a more prominent role in sustainable agriculture, reducing our reliance on chemical pesticides and promoting healthier ecosystems.

## 8. Conclusion

It is of utmost importance to identify antimicrobial compounds that are both ecologically sustainable and derived from natural sources in order to effectively manage plant diseases caused by microorganisms. The *Streptomyces* genus has been extensively researched and utilized as a biological control strategy against plant pathogenic bacteria. *Streptomyces* spp. show significant potential in current agricultural practices, such as their use as biofertilizers and for biological control, which may contribute to their continued dominance in the market economy. The biotechnological potential exhibited by these bacteria makes them highly promising microorganism for agricultural applications. However, it is crucial to carefully consider various unresolved issues in order to replicate the outcomes observed in controlled laboratory environments during extensive commercialization and field experiments. Therefore, the cultivation of *Streptomyces*-derived biological compounds is imperative for enhancing crop health and promoting sustainable agriculture.

## 9. Future perspective

*Streptomyces* spp. have shown significant promise as biological control agents for plant pathogens. These filamentous, Gram-positive bacteria are known for their ability to produce a wide range of secondary metabolites, including antibiotics and antifungal compounds, which can inhibit the growth of plant pathogens. Here are some future perspectives:

a. **Understanding Diversity:** Researchers are likely to continue exploring the diversity within *Streptomyces* species. Different strains may possess varying abilities to produce bioactive compounds, and understanding this diversity can lead to the discovery of more potent biological control agents.
b. **Metagenomics and Genomics:** Advances in metagenomics and genomics can aid in the identification and characterization of novel *Streptomyces* strains with enhanced biocontrol properties. These techniques can also help in understanding the genetic basis of antibiotic production in *Streptomyces.*
c. **Synthetic Biology:** Synthetic biology approaches may be employed to engineer *Streptomyces* strains for improved biocontrol capabilities. This could involve enhancing the production of specific antifungal or antibacterial compounds or optimizing their environmental survival and competitiveness.
d. **Eco-Friendly Formulations:** Researchers will likely work on developing eco-friendly formulations of *Streptomyces*-based biopesticides. This could involve creating stable formulations, improving shelf life, and ensuring ease of application.
e. **Integrated Pest Management (IPM):** *Streptomyces*-based biocontrol agents may become an integral part of IPM strategies. Combining biological control with other pest management approaches like crop rotation, organic farming practices, and resistant crop varieties can provide a more comprehensive solution.
f. **Regulatory Framework:** As *Streptomyces*-based products gain prominence, regulatory agencies may establish clearer guidelines and standards for their registration and use. Ensuring safety and efficacy will be crucial.

g. **Field Trials and Commercialization:** Large-scale field trials and commercialization efforts will be essential to bring *Streptomyces*-based biopesticides to the market. This may involve partnerships between research institutions, agribusinesses, and governmental agencies.
h. **Environmental Impact Assessment:** Continual assessment of the environmental impact of using *Streptomyces*-based biocontrol agents will be necessary. This includes evaluating their effects on non-target organisms and assessing their long-term sustainability.
i. **Education and Outreach:** Raising awareness among farmers and agricultural stakeholders about the benefits and proper use of *Streptomyces*-based biopesticides will be crucial for their adoption.
j. **Global Collaboration:** Given the global nature of agriculture and the spread of plant pathogens, international collaboration in research, development, and sharing of best practices will be important.

*Streptomyces* species hold great potential as biological control agents for plant pathogens, and ongoing research is likely to enhance their effectiveness, safety, and practicality for sustainable agriculture in the future.

## Author contributions

SK: Investigation, Methodology, Validation, Writing – original draft. SS: Investigation, Methodology, Data curation, Project administration, Writing – review and editing. AK: Conceptualization, Resources, Supervision, Visualization, Writing – review and editing. TM: Writing – review and editing, Data curation, Formal analysis.
